# Supplementary figures and images for: Long-read sequencing reveals complex patterns of wraparound transcription in polyomaviruses
Source: PLoS Pathog. 2022 Apr 1;18(4):e1010401. doi: 10.1371/journal.ppat.1010401 (PMC9007360; doi:10.1371/journal.ppat.1010401)

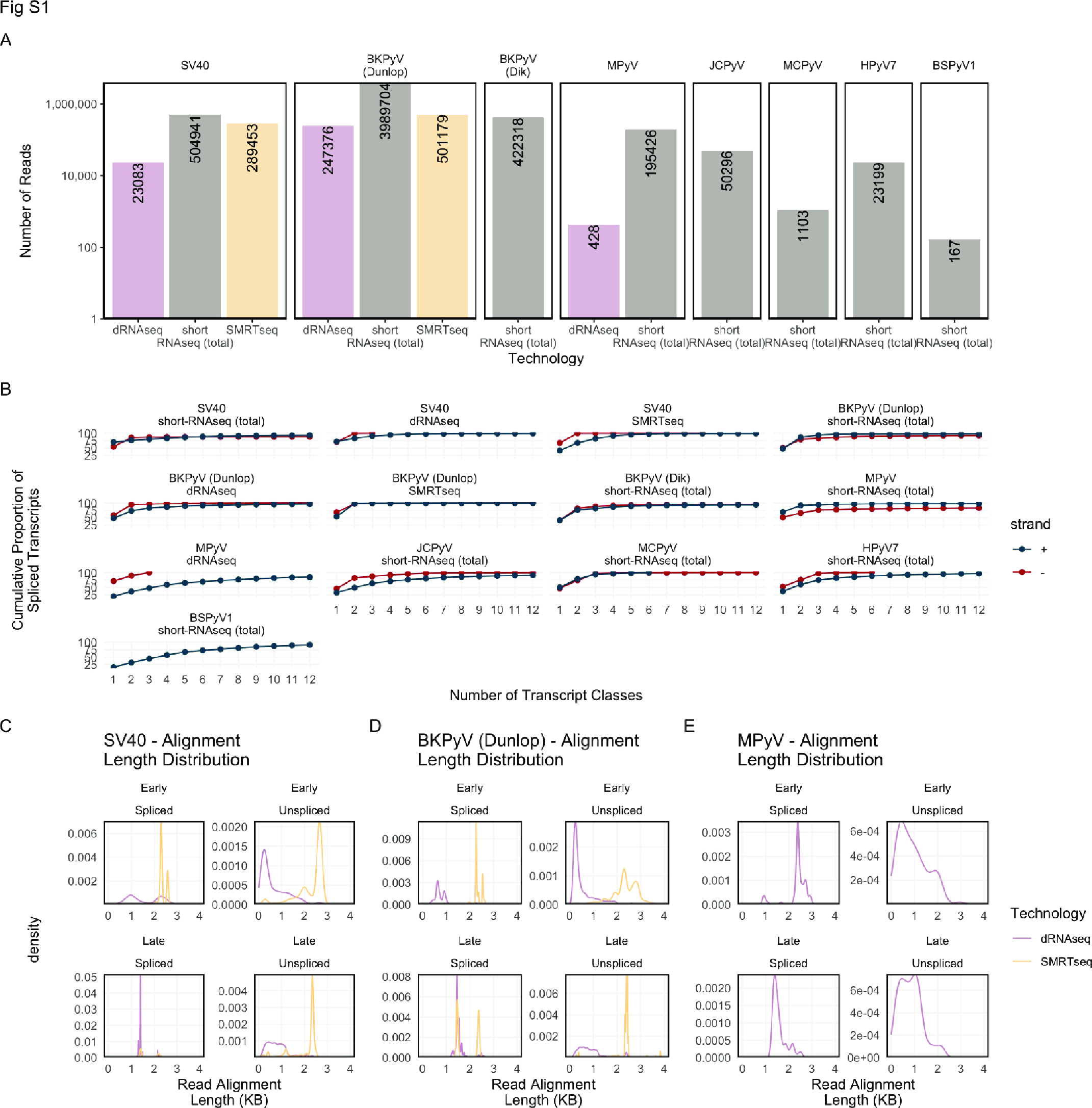

Supplement: S1 Fig — A. The number of reads for all datasets studied here. For long-read dRNAseq and SMRTseq, this number includes spliced and unspliced reads. Because short reads are only useful for transcript characterization when they span a splice junction, the counts for short-reads represent the number of splice-junction-spanning reads. B. The cumulative percentage of transcripts in each number of transcript classes, by strand. The X-axis indicated the total number of transcript classes. The Y axis indicates the cumulative percentage of transcripts within those transcript classes. These plots indicate that most transcripts in most samples are contained within the first few transcript classes. C-E. The alignment length distribution of early, late, spliced, and unspliced transcripts for dRNAseq and SMRTseq data from SV40 (C), BKPyV Dunlop (D), and MPyV (E). The X axis indicates the length of a read’s alignment, while the Y axis indicates the density/percentage of transcripts with a given alignment length. This plot shows that dRNAseq and SMRTseq data sample from RNA populations of different length. (TIFF) [file ppat.1010401.s003.tiff]

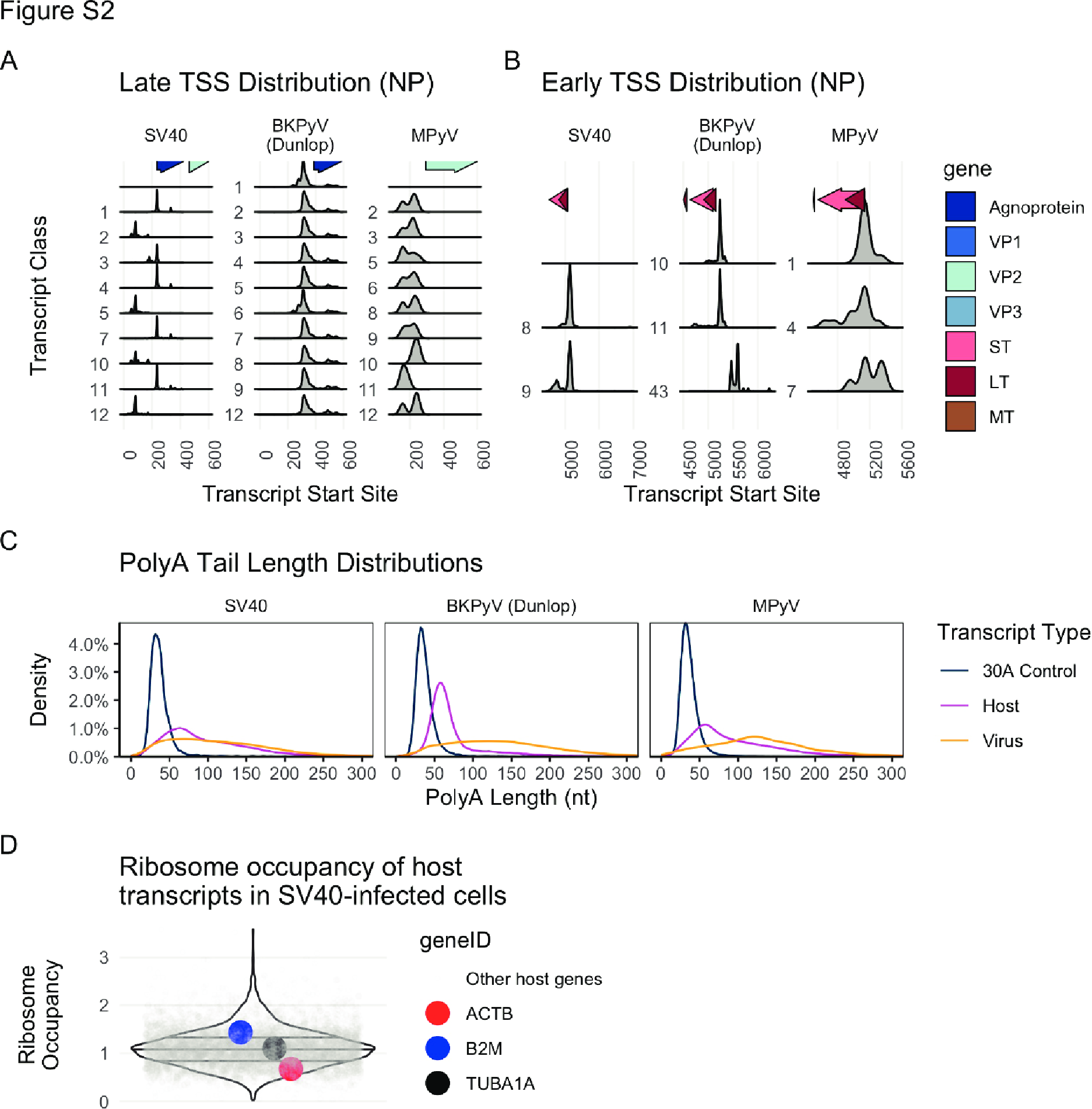

Supplement: S2 Fig — A, B. The distribution of transcript start sites for late (A) and early (B) transcripts for SV40 (left column), BKPyV Dunlop (middle column), and MPyV (right column). The arrows indicate the viral ORF positions. C. The distribution of polyA tail lengths for the 30-adenine ENO2 control (black), host (red), and viral (yellow) transcripts for SV40, BKPyV Dunlop, and MPyV. The X axis indicates the length of the polyA tail, while the Y axis indicates the density/percentage of transcripts with each length. D. Ribosome occupancy of host transcripts in SV40-infected cells. Each grey dot is a host transcript. The red, blue, and black dots are specifically noted host transcripts. Ribosome occupancy is on the Y axis, while the X axis does not hold value. Lines on the violin plot indicate 1st, 2nd, and 3rd quartiles. (TIFF) [file ppat.1010401.s004.tiff]

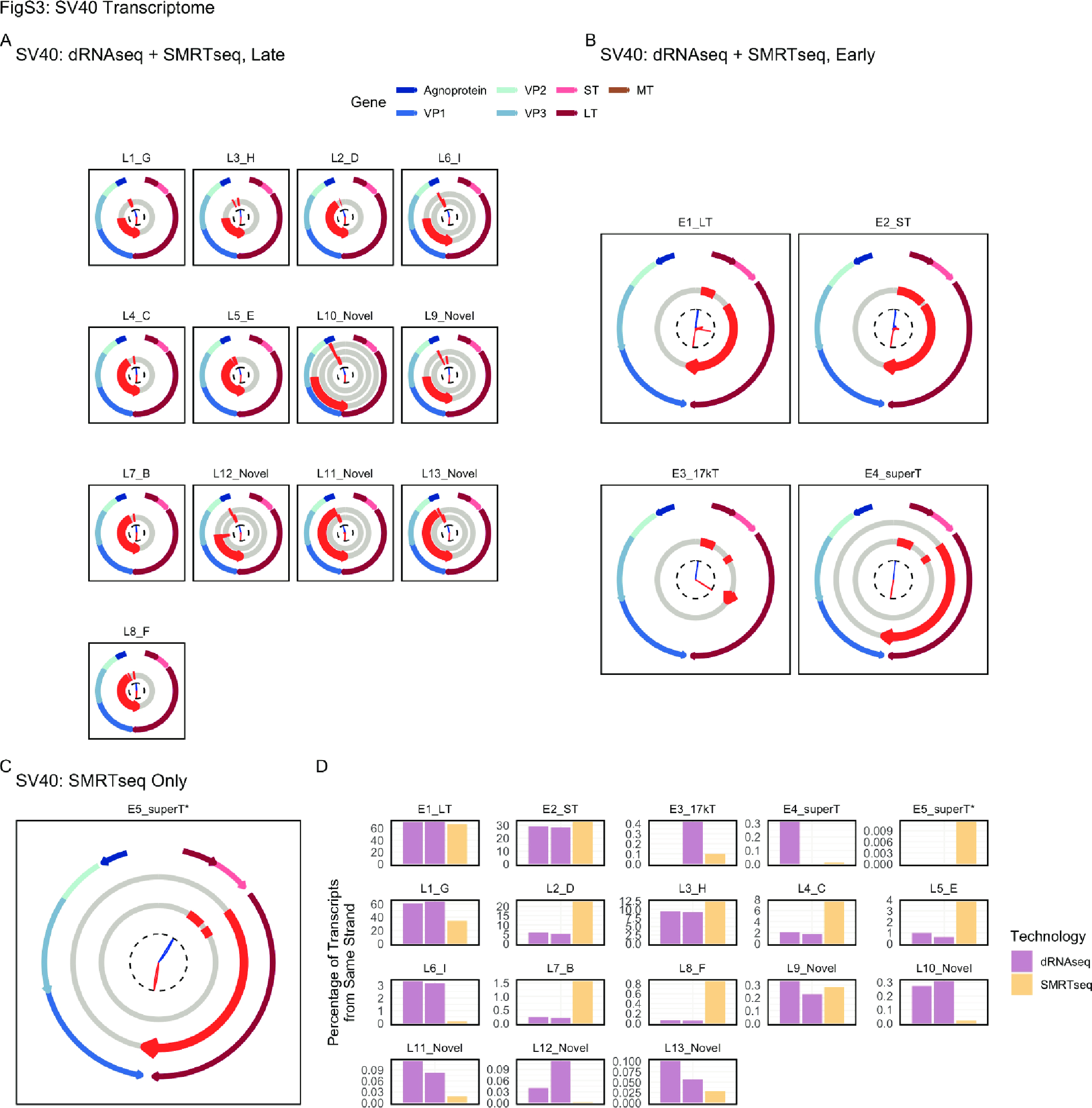

Supplement: S3 Fig — A-C. Watch plots indicating all identified transcripts in SV40. (A) and (B) show transcripts that were identified in both dRNAseq and SMRTseq data, while (C) shows transcripts identified in SMRTseq only. Pre-mRNA paths are not drawn but can be inferred as indicated in Fig 4. D. Barplots that show the abundance of each transcript type in the dRNAseq and SMRTseq data. Here, there are two dRNAseq bars (one per replicate). The Y axis indicates the percentage of transcripts of the same strand. As discussed in the methods, alignment of superT and superT* was challenging, so the actual abundance of these transcripts is higher than reported here. (TIFF) [file ppat.1010401.s005.tiff]

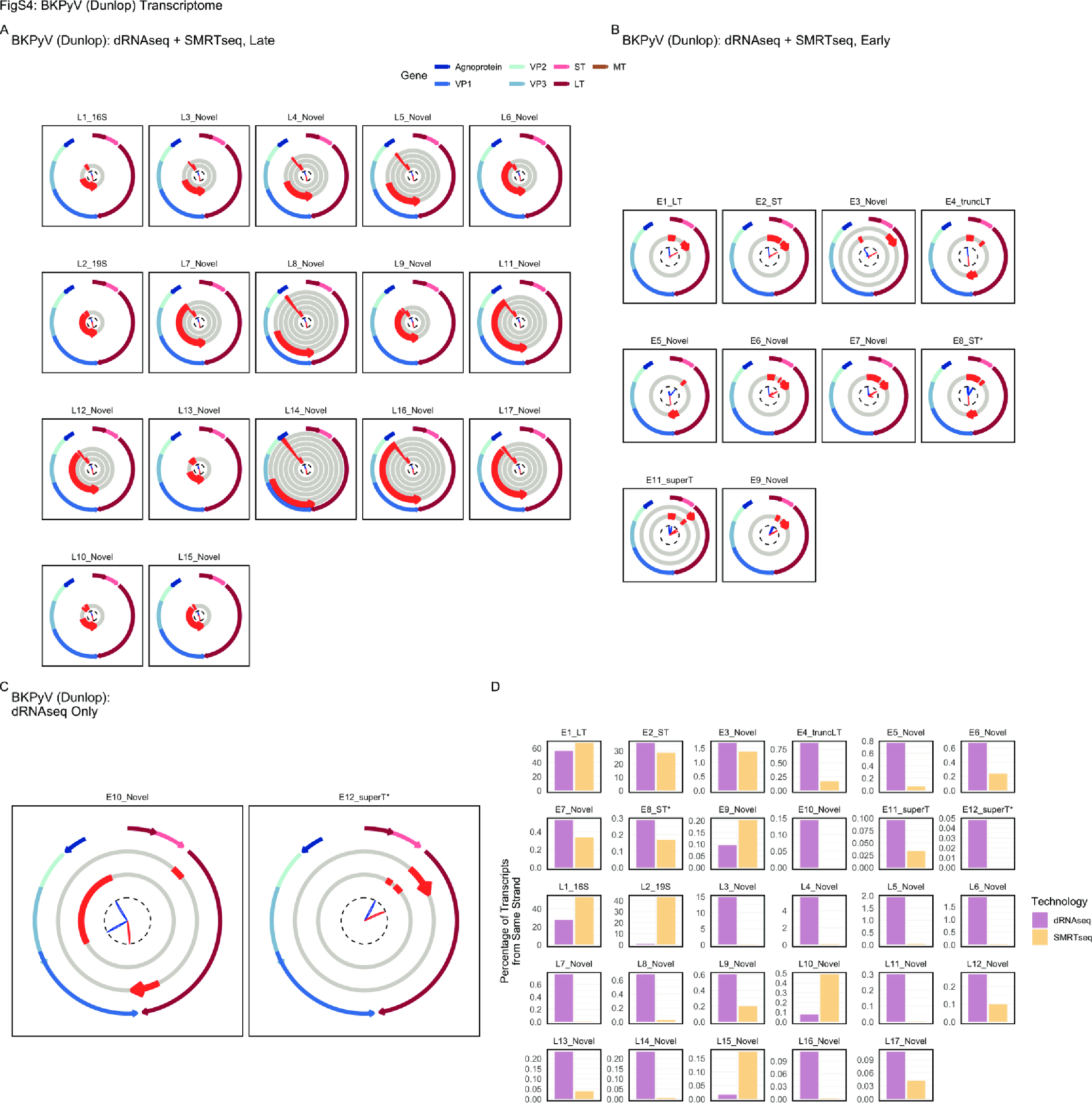

Supplement: S4 Fig — A-C. Watch plots indicating all identified transcripts in BKPyV Dunlop. (A) and (B) show transcripts that were identified in both dRNAseq and SMRTseq data, while (C) shows transcripts identified in dRNAseq only. Pre-mRNA paths are not drawn but can be inferred as indicated in Fig 4. D. Barplots that show the abundance of each transcript type in the dRNAseq and SMRTseq data. The Y axis indicates the percentage of transcripts of the same strand. As discussed in the methods, alignment of superT and superT* was challenging, so the actual abundance of these transcripts is higher than reported here. (TIFF) [file ppat.1010401.s006.tiff]

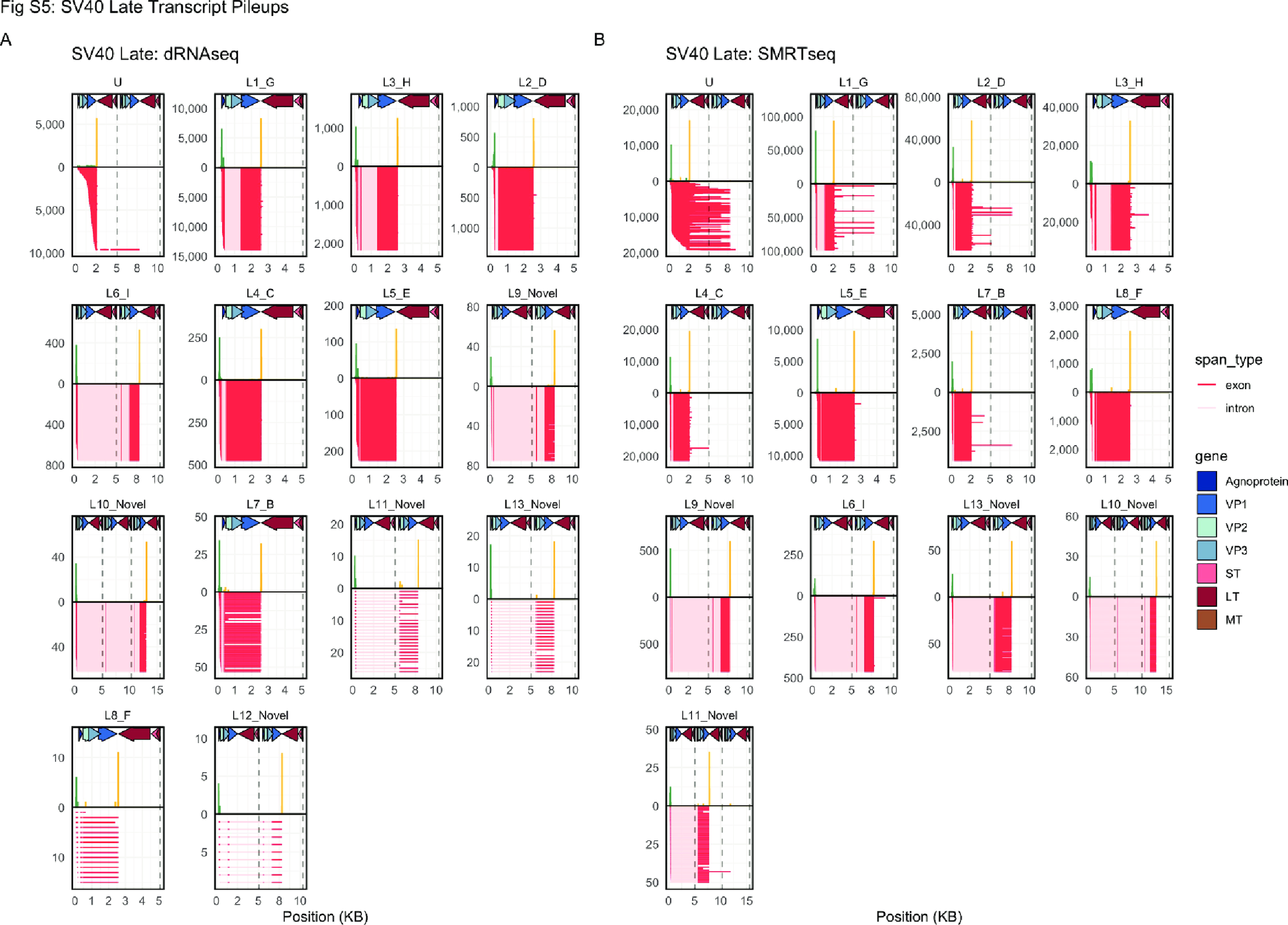

Supplement: S5 Fig — A, B. Read pileups showing the late transcripts identified in SV40 dRNAseq (A) and SMRTseq (B). The arrows at the top of the plot indicate the viral ORF positions. Below the X axis, each row is an individual transcript, with exons indicated in red and splice junctions/introns indicated in pink. Above the X axis are histograms indicating the transcript start (teal) and transcript end (yellow) sites. (U: unspliced). Transcript classes are ordered in order of decreasing abundance in each dRNAseq or SMRTseq dataset. (TIFF) [file ppat.1010401.s007.tiff]

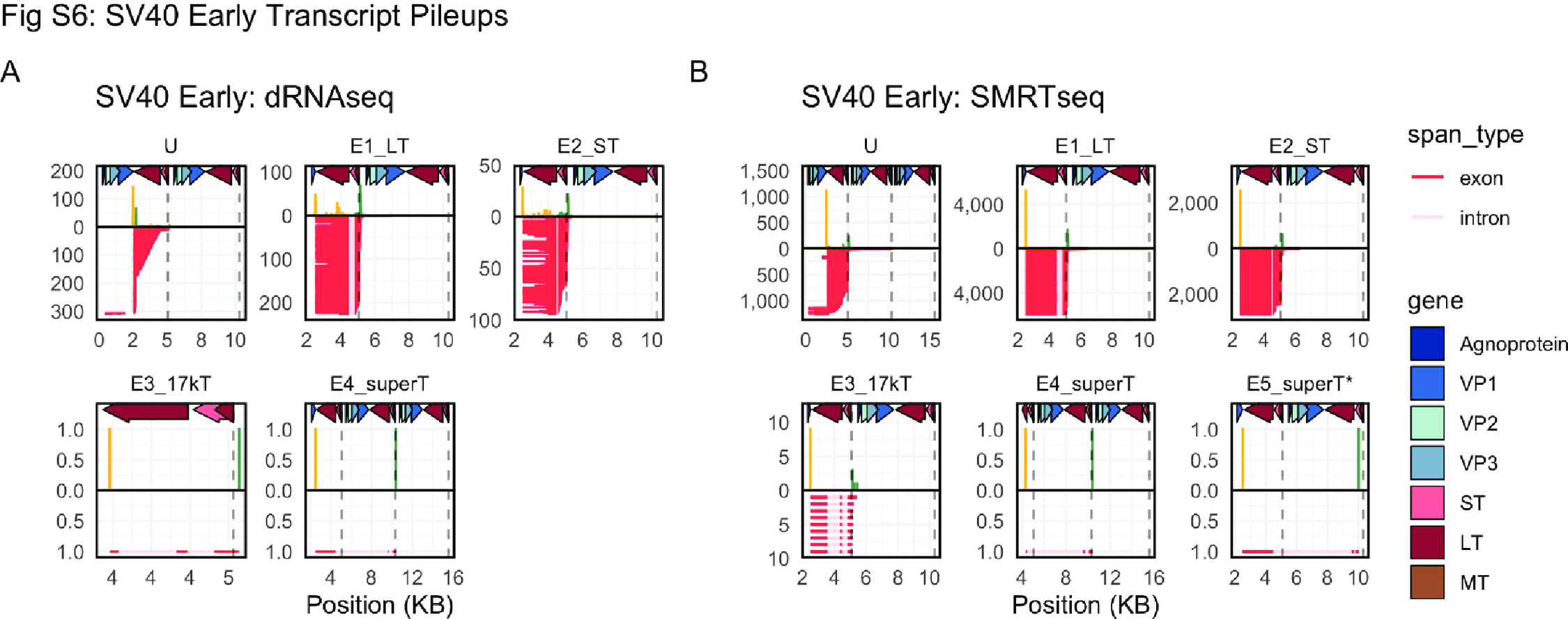

Supplement: S6 Fig — A, B. Read pileups showing the early transcripts identified in SV40 dRNAseq (A) and SMRTseq (B). The arrows at the top of the plot indicate the viral ORF positions. Below the X axis, each row is an individual transcript, with exons indicated in red and splice junctions/introns indicated in pink. Above the X axis are histograms indicating the transcript start (teal) and transcript end (yellow) sites. (U: unspliced). Transcript classes are ordered in order of decreasing abundance in each dRNAseq or SMRTseq dataset. (TIFF) [file ppat.1010401.s008.tiff]

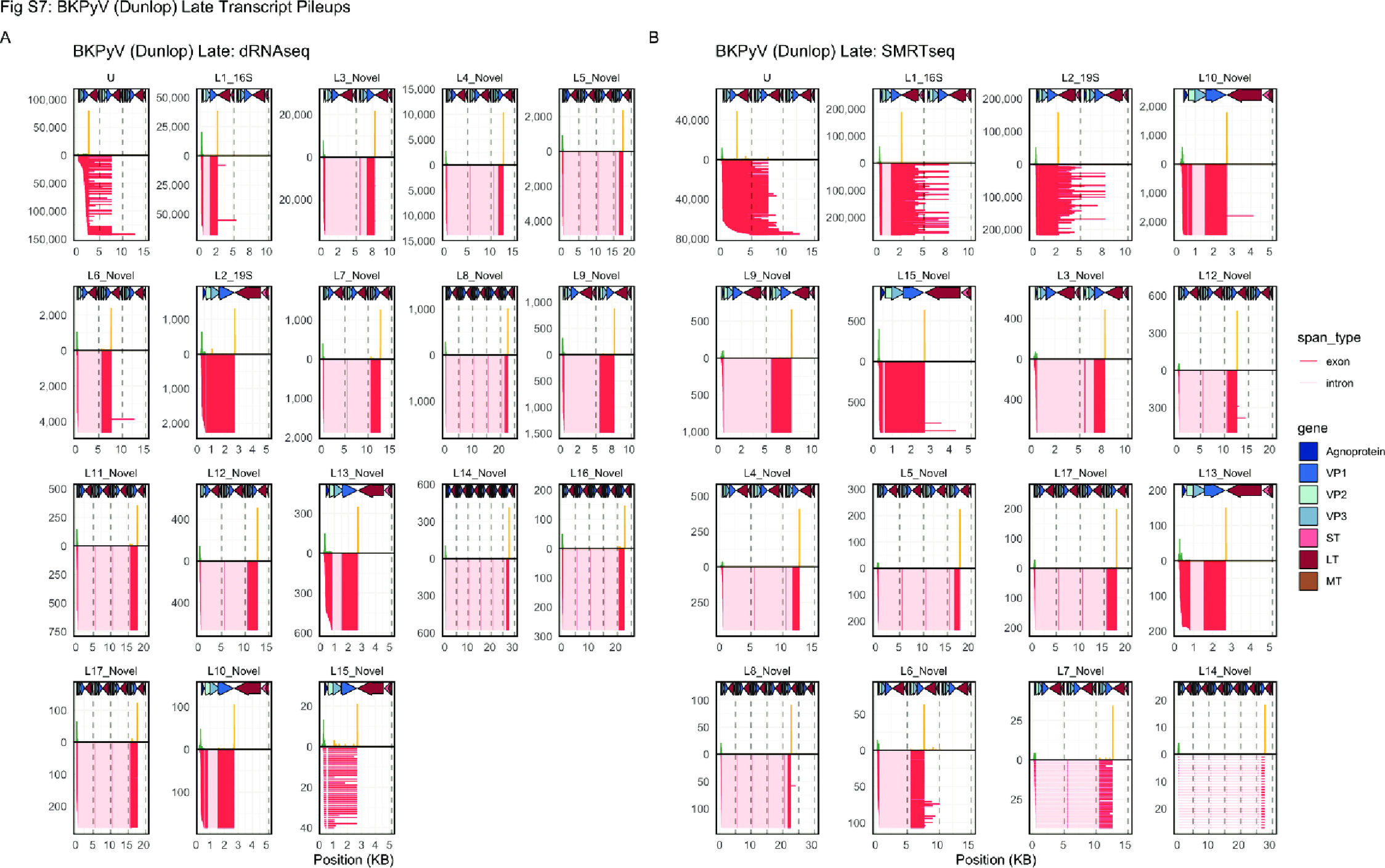

Supplement: S7 Fig — A, B. Read pileups showing the late transcripts identified in BKPyV Dunlop dRNAseq (A) and SMRTseq (B). The arrows at the top of the plot indicate the viral ORF positions. Below the X axis, each row is an individual transcript, with exons indicated in red and splice junctions/introns indicated in pink. Above the X axis are histograms indicating the transcript start (teal) and transcript end (yellow) sites. (U: unspliced). Transcript classes are ordered in order of decreasing abundance in each dRNAseq or SMRTseq dataset. (TIFF) [file ppat.1010401.s009.tiff]

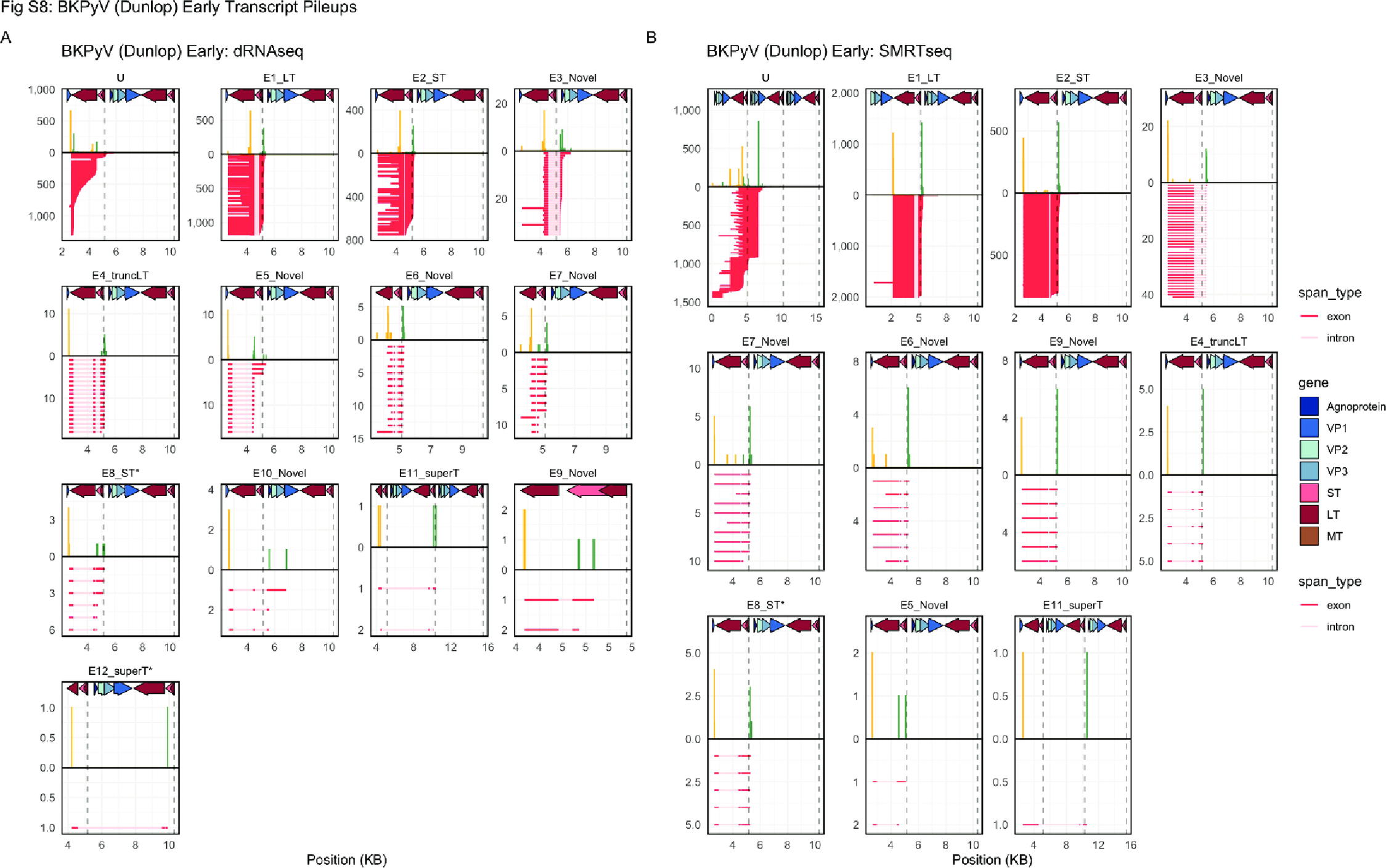

Supplement: S8 Fig — A, B. Read pileups showing the early transcripts identified in BKPyV Dunlop dRNAseq (A) and SMRTseq (B). The arrows at the top of the plot indicate the viral ORF positions. Below the X axis, each row is an individual transcript, with exons indicated in red and splice junctions/introns indicated in pink. Above the X axis are histograms indicating the transcript start (teal) and transcript end (yellow) sites. (U: unspliced). Transcript classes are ordered in order of decreasing abundance in each dRNAseq or SMRTseq dataset. (TIFF) [file ppat.1010401.s010.tiff]

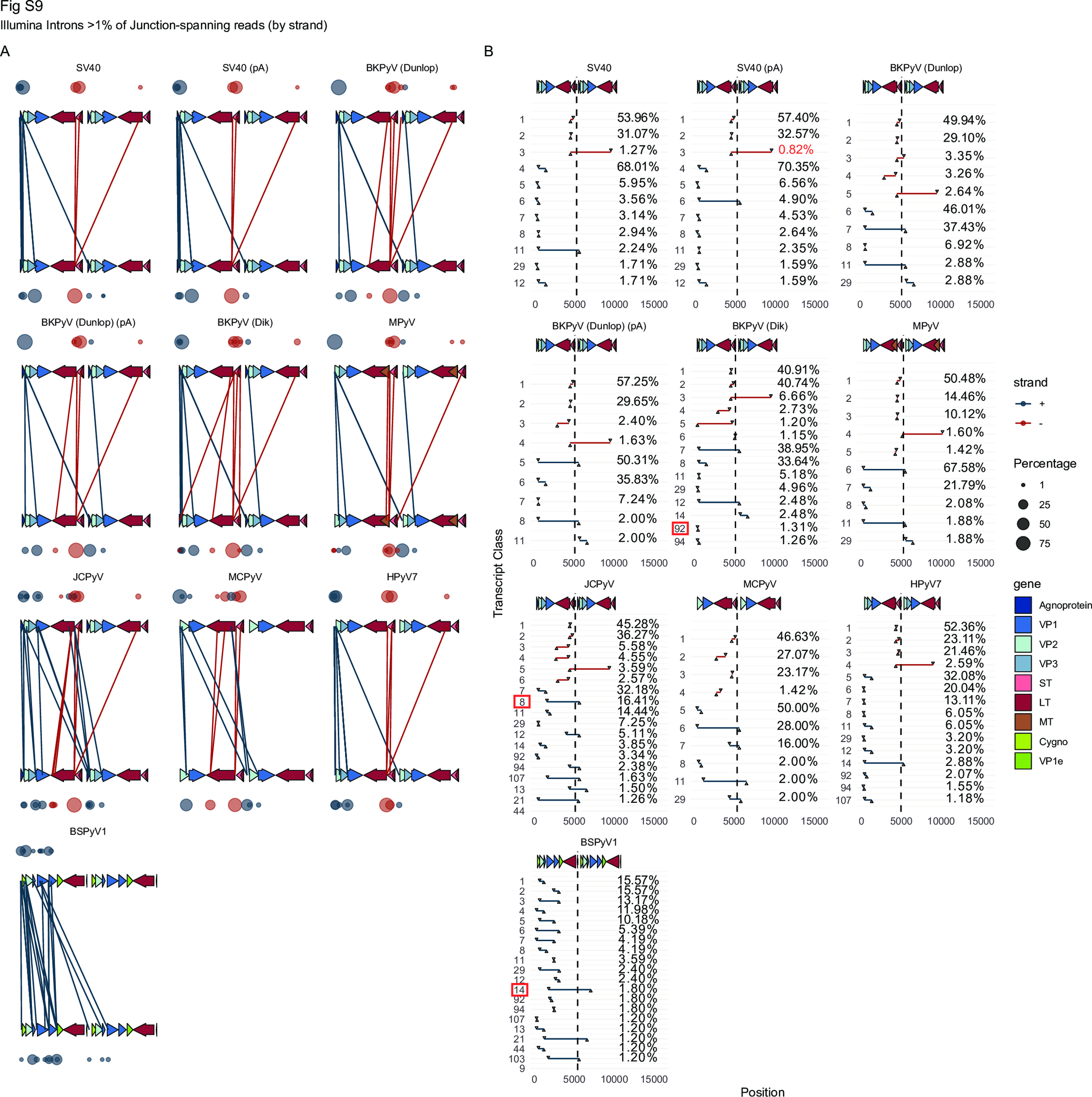

Supplement: S9 Fig — A. Intron plots generated from short-read RNAseq. The arrows at the top and bottom of each panel indicate the position of viral ORFs. The lines indicate specific introns identified in the RNAseq data, with the 5’ end on the top and the 3’ end on the bottom. The blue color indicates late transcripts, with red indicating early transcripts. The size of the circles above and below the viral ORF maps indicate the percentage of junction-spanning reads with a 5’ end (on top) or 3’ end (on bottom) at that position. Junctions are plotted if they are at least 1% of early or late transcripts, except for the SV40 pA superT junction (transcript class 3) which is just below threshold but is of interest. B. Another representation of intron plots for each virus. The top arrows indicate the position of viral ORFs. The X axis indicates the genomic position for each splice. The Y axis indicates a single transcript class, with that class’ intron plotted as a line. The percentage of early or late transcripts is indicated with the numeric percentage. Junctions are plotted if they are at least 1% of early or late transcripts, except for the SV40 pA superT junction (transcript class 3) which is just below threshold but is of interest. Transcript classes with labels surrounded by a red box indicate that they lack either the 5’ GT (donor) or 3’ AG (acceptor) splice site dinucleotides. (TIFF) [file ppat.1010401.s011.tiff]

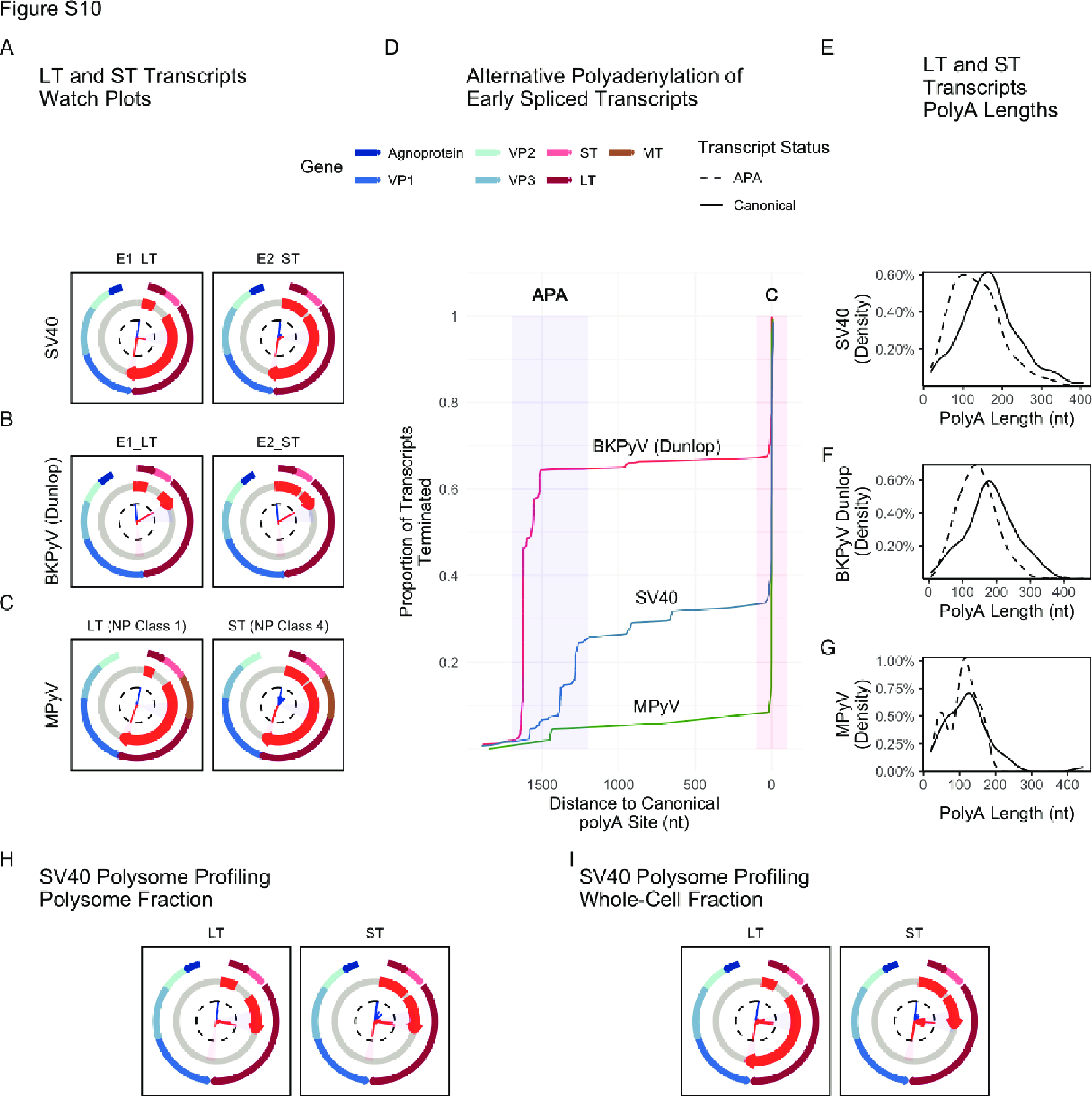

Supplement: S10 Fig — A-C. Watch plots indicating the LT and ST transcripts for SV40 (A), BKPyV Dunlop (B), and MPyV (C). The focus of these plots is the distribution of transcript end positions, which are the inner red arms. The region of APA of highlighted in blue, with the canonical transcript end sites highlighted in red. D. A cumulative incidence plot of transcript termination in SV40 (blue), BKPyV Dunlop (red), and MPyV (green). The X axis indicates the distance to the canonical polyA site, while the Y axis indicates the percentage of transcripts that have terminated by that position. E-G. Density plots showing the distribution of polyA tail lengths for LT and ST transcripts that end at the canonical site (solid) or undergo APA (dashed) for SV40 (E), BKPyV Dunlop (F), and MPyV (G). The x axis indicates the length of the polyA tail, while the Y axis indicates the density/proportion of transcripts with the given length. H-I. Watch plots indicating the LT and ST transcripts from polysome-associated (H) or whole-cell (I) RNAs. The focus of these plots is the distribution of transcript end positions, which are the inner red arms. The region of APA of highlighted in blue, with the canonical transcript end sites highlighted in red. (TIFF) [file ppat.1010401.s012.tiff]

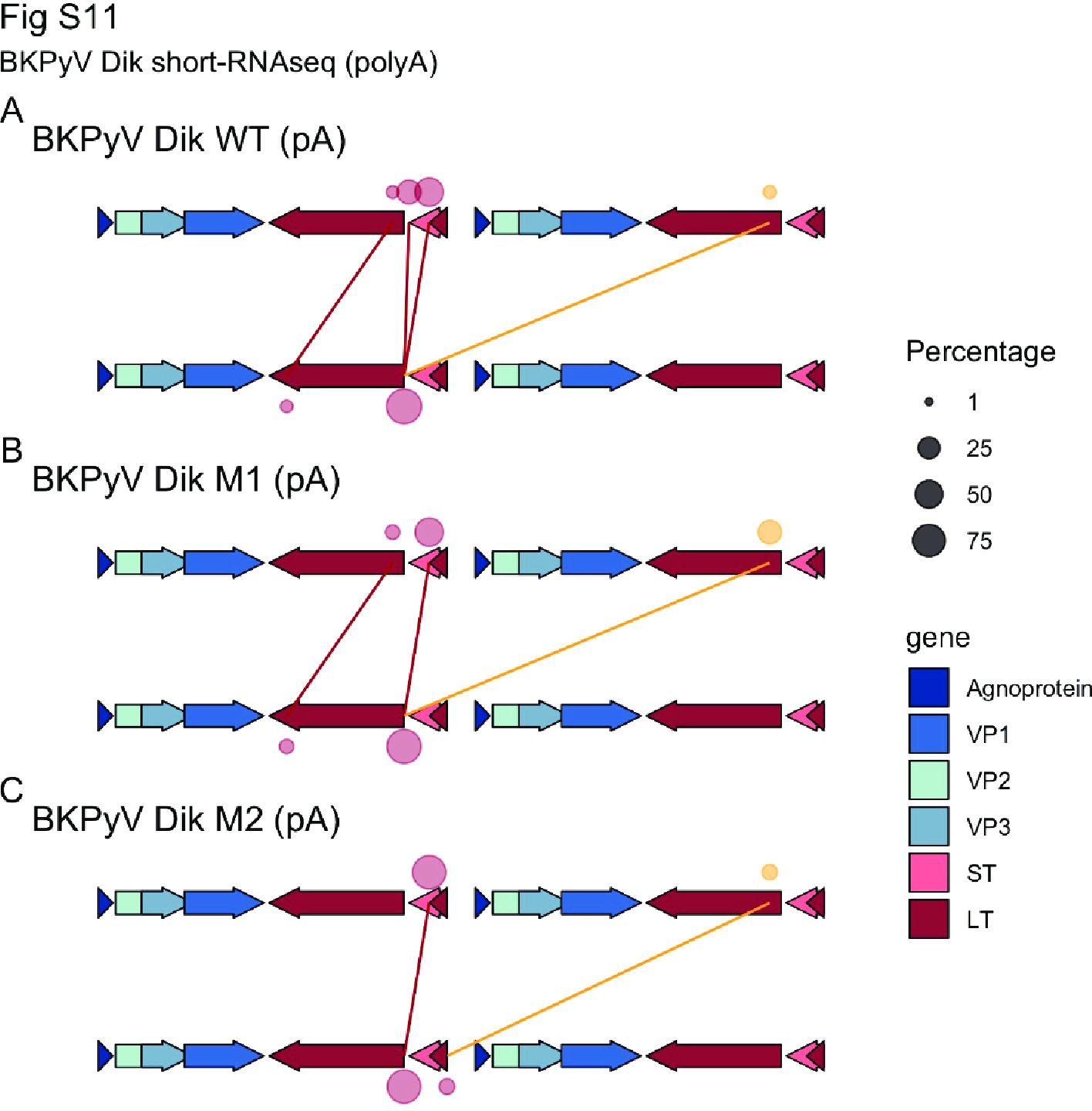

Supplement: S11 Fig — A-C. Intron plots generated from short-read (polyA) RNAseq of cells infected with BKPyV WT, or the M1 or M2 mutants. The arrows at the top and bottom of each panel indicate the position of viral ORFs relative to the standard BKPyV genome—note that the genomes of mutants M1 and M2 are altered as indicated in Fig 5E. The lines indicate specific introns identified in the RNAseq data, with the 5’ end on the top and the 3’ end on the bottom. The size of the circles above and below the viral ORF maps indicate the percentage of junction-spanning reads with a 5’ end (on top) or 3’ end (on bottom) at that position. Only early junctions that are at least 1% of early transcripts are plotted. The superT junction is colored in gold. (A) Intron plot for BKPyV Dik WT. (B) Intron plot for BKPyV Dik M1. (C) Intron plot for BKPyV Dik M2. (TIFF) [file ppat.1010401.s013.tiff]

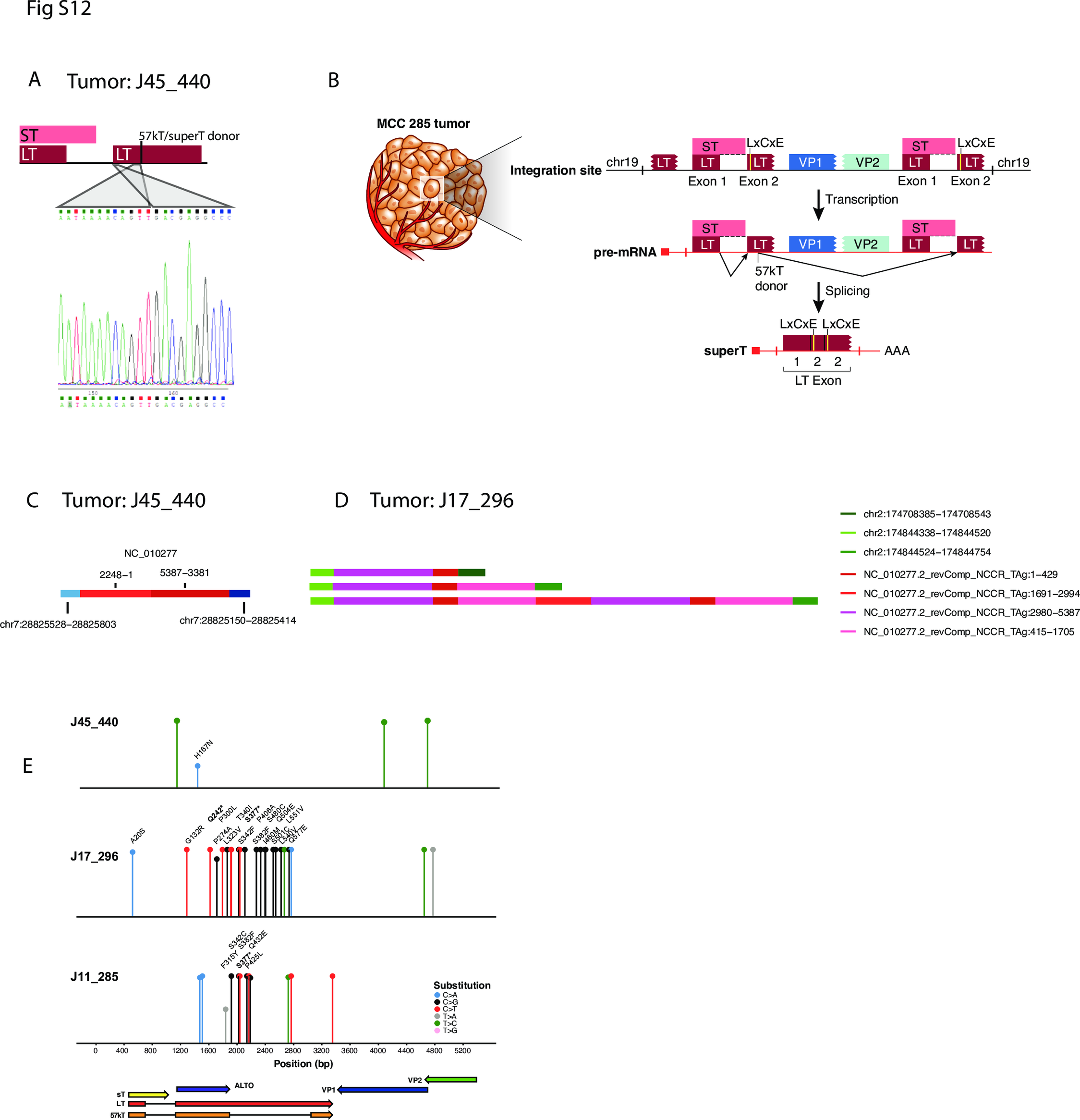

Supplement: S12 Fig — A. Sanger sequencing of an RT-PCR product from MCC J45_440, showing the superT-specific junction. B. A schematic detailing the MCC 285 MCPyV integration site, showing how it is possible that superT is generated via cis-splicing. C. The assembled viral block in MCC tumor J45_440. This integration site is based on de-novo assembly using short whole genome sequencing reads. Despite only assembling one viral block, we found that 1) there are likely 2 copies of the viral genome, and 2) the 5’ viral integration site appears to fall on chromosome 7 “after” the 3’ viral integration site, observations consistent with the existence of two copies of the viral genome in tandem separated by a small segment of host DNA at this integration site. D. The assembled viral blocks in MCC tumor J17_296. The longest block contains two copies of the early region. E. Lollipop plots showing identified SNPs in the MCPyV genomes of J45_440, J17_296, and J11_285. The gene-map below the Fig indicates the position of viral ORFs. Each lollipop is colored according to the nucleotide substitution identified. (TIFF) [file ppat.1010401.s014.tiff]
