# Supplementary material for: Long-read sequencing reveals complex patterns of wraparound transcription in polyomaviruses
Source: PLoS Pathog. 2022 Apr 1;18(4):e1010401. doi: 10.1371/journal.ppat.1010401 (PMC9007360; doi:10.1371/journal.ppat.1010401)
Supplement: Attachment — Submitted filename: PyV_transcriptome_response_to_reviewers.pdf [file ppat.1010401.s015.pdf]

Reviewer 1

Nomburg et al have provided a comprehensive analysis of transcription patterns in diverse polyomaviruses. The care and detail applied to this work represents a real tour de force and offers a range of exciting insights into the biology of these underappreciated pathogens. While the general transcriptional analysis is compelling, I was particularly intrigued by the analysis of SuperT, which really served to highlight the power of the methodology. Overall, the manuscript is well written and the reams of supporting data are readily accessible. As such, I only have a few significant critiques/comments, which I think would improve the manuscript, as well as a number of minor comments. Assuming these are addressed then I would highly recommend this manuscript for acceptance.

Thank you very much for your positive comments.

Major comments:

There are no additional key experiments required from my perspective. However, I do think it is imperative that the following analyses are performed or, if not possible, appropriately caveated in the text. It is also imperative to ensure all sequencing data (inc. raw fast5 files) are made available at the time of publication.

• From the bioproject page, it appears only the FASTQ datasets derived from Nanopore DRS have been uploaded. Given the rapid improvements in basecalling associated with nanopore, the authors should really upload the raw fast5 data. This can be done via the European Nucleotide Archive in a relatively simple manner (make a tarball of the raw fast5 data and upload) and should be included under the bioproject ID.

The fast5 data have been uploaded to the SRA under BioProject PRJNA806471. Furthermore, we have lifted the embargo from the fastq data, which are present at BioProject PRJNA780012.

• One element that I think is lacking from the results is a clear analysis of how abundant each transcript is. While I kind roughly estimate this for SV40 from Figure 1G, it would be very useful to have this information for all the viruses. Unsurprisingly there is a large dynamic range and it looks like novel transcripts are generally not very abundant but this is not always clear.

Thank you for this comment. We have added plots indicating the relative abundance of each transcript in dRNAseq data as Figure 2B and Figure 3B, below the relevant transcript map.

• Some further analysis of the polysome sequencing datasets is warranted. It would be particularly interesting to know whether transcripts originating from wraparound transcription are also translated and if so, how their loading compares to raw abundance.

We have now added stars to Figure 1G to indicate which transcripts are wraparound transcripts, and have added the following text to the manuscript:

LINE 186: “Notably, we find that there is limited difference in the polysome and whole-cell relative abundance of wraparound transcripts (**Figure 1G**), suggesting that leader-leader splicing has limited effect on translation.”

• How many of the described features (e.g. APA, wraparound transcription) are temporally regulated? Do these only arise during Late stages or would they also be expected at early timepoints? i.e. can the authors justify not profiling multiple distinct timepoints during infection.

Thank you for this comment. Our goal was to favor breadth of viruses over additional timepoints, as our primary goal was transcript identification. However, to address your question we analyzed publicly available temporal data for BKPyV/JCPyV (from Asssetta et al., with timepoints of 3, 6, and 9 days post infection) and MPyV (from Garren et al., with timepoints of 18, 24, and 36 hours post infection) to investigate changes in the cumulative proportion of spliced reads that indicate wraparound transcription over time (plot is below).

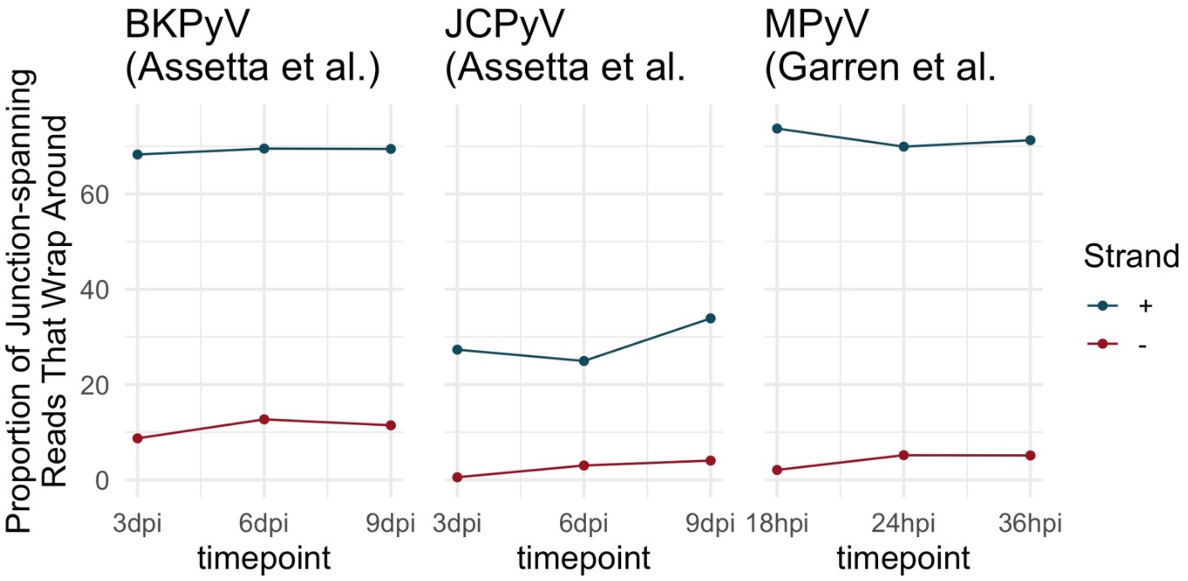

We find that there were only small changes in relative abundance of wraparound transcripts over time.

Because we do not otherwise use the Assetta et al. BKPyV data (we only use their JCPyV-9dpi data and use our own BKPyV data) and only use the 36hpi timepoint from Garren et al., to avoid any confusion we have opted not to include these analyses in the manuscript. We do add the following note to the discussion:

LINE 378: “Finally, analysis of publicly-available temporal RNAseq data from JCPyV, BKPyV, and MPyV infection [16, 37] suggests limited changes in wraparound abundance over time. ”

Regarding APA, we do suspect that there could be temporal regulation since MPyV, despite containing the strongest premature polyA motif (AATAAA), has lowest incidence

of APA. This could be explained by the fact that our MPyV dRNAseq data came from cells at a much earlier phase of infection than the SV40 and BKPyV dRNAseq data. We plan to investigate this in future studies. We have added the following to the text:

LINE 384: “We observe less early transcript APA in MPyV despite presence of a canonical AATAAA polyadenylation motif. Notably, the MPyV dRNAseq data is derived from cells at a relatively early stage of infection, raising the possibility that there is temporal regulation of early transcript APA. Future studies are required to investigate the factors influencing APA incidence in polyomaviruses.”

Minor comments:

• Figures 2 & 3 are beautifully constructed but I could not adequately distinguish between Acceptor (pink?) and Exon (red). I would suggest changing one of those colors to better distinguish these features.

Thank you for the suggestion. We changed the acceptor lines to black.

• Lines 57-63 in the introduction would benefit from a few citations.

Done. We added references to (4) Fields BN. Fields' virology: Lippincott Williams & Wilkins; 2007 and to (5) DeCaprio JA, Garcea RL. A cornucopia of human polyomaviruses. Nature Reviews Microbiology. 2013;11(4):264-76.

• Line 130 – I think this should refer to Figure S2?

We have corrected this to refer to the BKPyV transcript map (Fig 3A).

• Figure S9 would benefit from an analysis of splice donor and acceptor motifs. It would be useful to then correlate rare/non-canonical motifs against transcript abundance.

Thank you for the suggestion. We have found that almost all observed splice events that are high enough abundance to be plotted use the conserved 5’ GT (donor) or 3’ AG (acceptor) splice site dinucleotides. We have indicated the three exceptions with red boxes in Figure S9.

• ‘In contrast’ is used frequently but is not always grammatically correct. Consider also using ‘By contrast’ where appropriate.

Thank you, we have corrected this.

• It’s not clear to me why the authors utilized the NEB poly(A) selection approach given it’s limited loading capacity? Using a larger scale poly(A) purification approach (e.g. Invitrogen Dynabeads) would obviate the need for vacuum-based concentration.

This is because our pilot experiment used the cheaper NEB kit, and we stayed with it because we knew it worked. Thank you for pointing this out - in our future work we’ll likely switch to the Invitrogen kit.

• Can the authors confirm that all transcript isoforms observed contained measurable

poly(A) tails? It is often beneficial to omit DRS reads without defined poly(A) tails (as reported by nanopolish) to reduce potential artefacts.

For our main analyses (with the exception of the APA tail length analyses in Figure S10E,F,G), we did *not* do any filtering based on nanopolish polyA tail information. To address your concern, we went back and checked if abundance or APA incidence changes when we first filter for transcripts with a Nanopolish “PASS” and a polyA tail of at least 30 adenines.

The two figures below show transcript abundance in the original (Unfiltered) and Nanopolish PASS/>30adenine tail filtered (pA_filtered) data. Notably, there is no difference in transcript abundance. (Note - The SV40 figure below slightly differs from Fig 2B because the one below contains just one replicate).

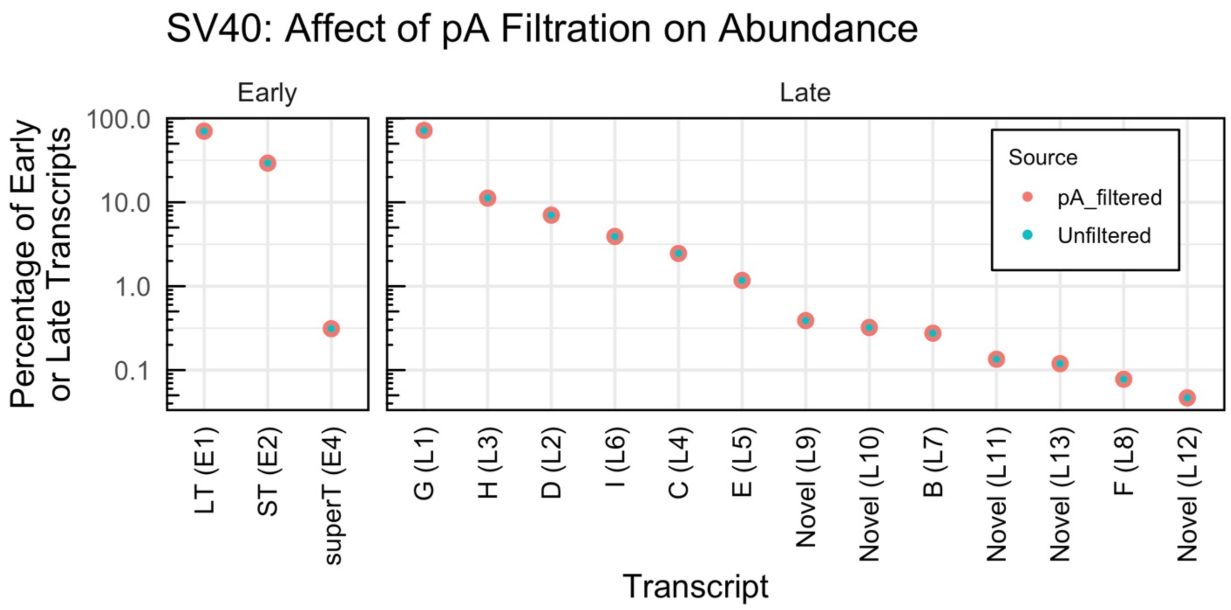

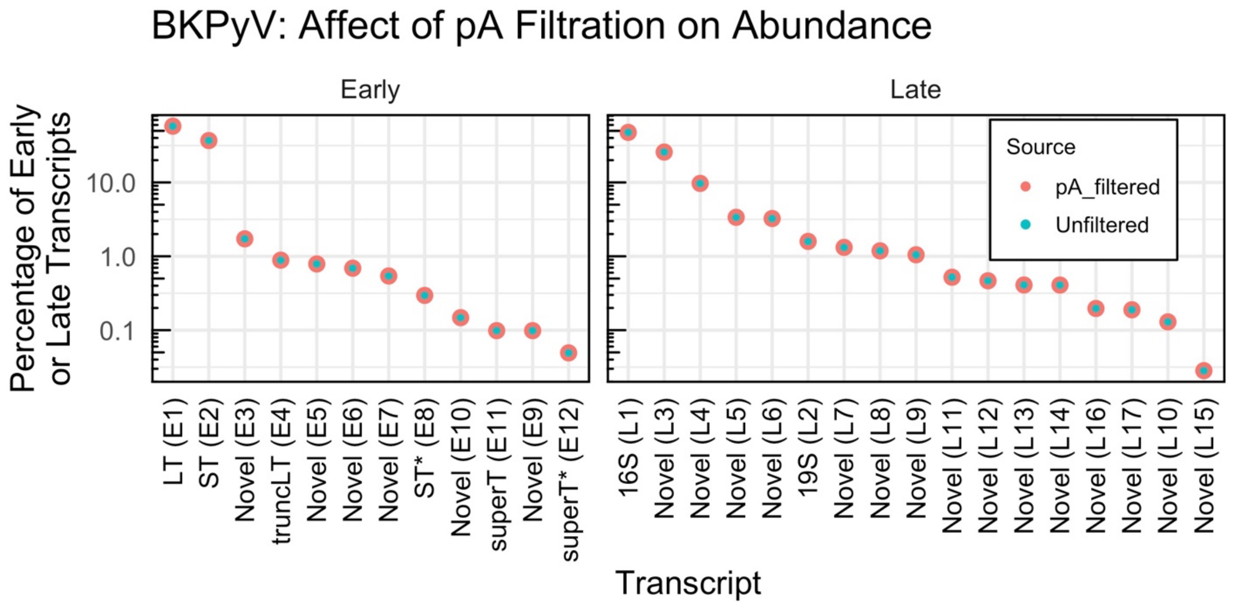

The next figure is a repeat of the APA analysis in Figure S10D but using data that has been filtered for Nanoplish PASS and 30adenine minimum polyA tail length. There is very little difference in the result compared to Figure S10D.

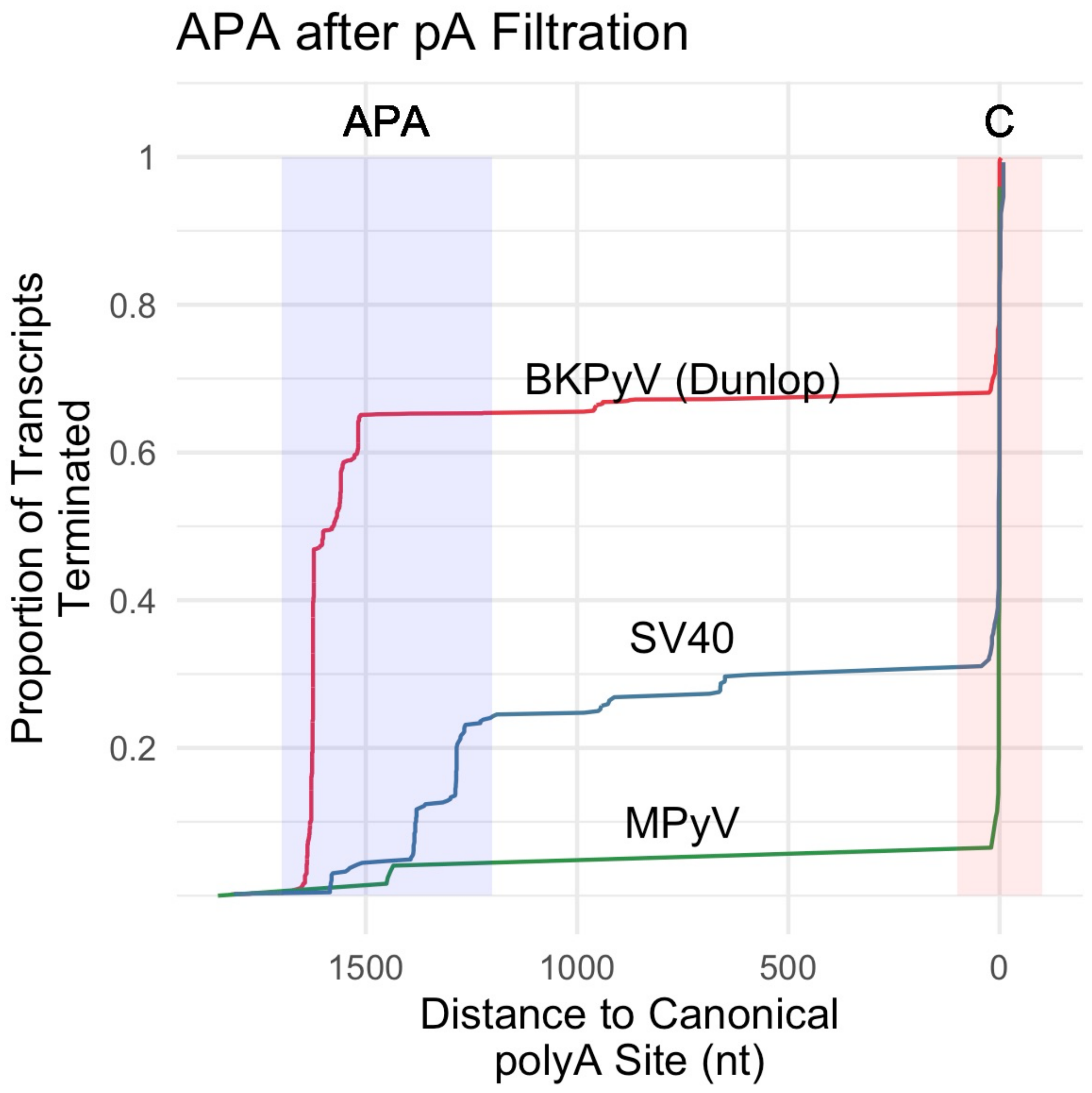

Thus, we have opted to leave our results as-is, without filtering based on Nanopolish, because filtering doesn't influence our results.

• It would help to order the transcript boxes shown in Fig S5A-B so that they occupy the same relative space (e.g. lower left should be L11_Novel in both A&B) as this makes visual comparison between DRS and PacBio results much easier.

Unfortunately this change would be challenging because not all transcript classes are observed in both dRNAseq and SMRTseq data. Currently, the transcript classes are ordered based on abundance in each respective dRNAseq or SMRTseq dataset. If we were to orient each box in the same relative position, we would have to introduce blank spaces, probably reduce the overall size of each box, and we wouldn't be able to order

by abundance. For these reasons, we feel we should leave the order of the transcript classes as-is.

I'm also not sure what to make of the 'read-through' reads observed in several of the PacBio samples (e.g. U, L1_G)? Is this an artefact?

We suspect these are transcripts that are intermediate and haven't been fully spliced! We don't think this is an artifact. We do point out that the lines of individual reads/rows in each plot are deceptively thick, so there aren't that many unspliced wraparound transcripts as there appears - compare the observed wraparound transcript lines vs the yellow histogram of end sites (e.g. in Figure S5B U). This is the major weakness of our pileup plots, as thinner lines would make the entire plot harder to see. We mention this limitation in the methods at line 818.

I also find the use of red (TSS) and blue (PAS) to be slightly confusing in the context of also showing ORFs above in red and blue.

Thank you for the suggestion. We have changed the read pileup start and end histograms to teal and yellow.

• It might be a simple formatting issues but I do not see any of the pre-mRNA paths shown on any of the watch plots except for the legend schematic in Fig4). While this might also be intentional (i.e. I can roughly infer this for each plot), it would be useful to have those pre-mRNA paths shown on each individual plot.

This was indeed intentional, as pre-mRNA paths must be manually drawn, and our intention was indeed that they are inferred. Based on your feedback we have added them to the watch plots in Figure 4. We have not included them in the supplementary figures, but now mention in the figure legends for Figure S3 and S4 that the pre-mRNA paths can be inferred as indicated by Figure 4.

Reviewer 2

This is an excellent transcriptomic study performed Nomburg et al. on multiple diverse polyomaviruses. The authors use a combination of long and short format high throughput sequencing of polyA-enriched RNAs. The combination of techniques combined with the clever application of evolutionary conservation makes for one of the most diverse comprehensive evolutionary comparative study of viral transcriptomics I am aware of. The authors use both of these traits (detection in alternative methods and/or evolutionary conservation) to identify new transcripts. They then confirm that at least some of these newly identified transcripts are likely translated since they are found in polysomal-enriched fractions of RNA. Although no function is provided for the newly identified transcripts, the fact that some are found on polysomes serve as useful hypothesis generators worthy of future study. Overall, this work will likely be a useful study to many polyoma virologists for years to come.

Thank you very much for your positive comments.

Major comment:

I could not find reference to important Acheson paper that first described the phenomena of "giant RNAs", or wrap around transcripts further elucidated here. Minimally, this needs to be cited (I apologize if I missed it somehow), and I suggest that it warrants a sentence or two in the Intro and Discussion: Acheson NH. Polyoma virus giant RNAs contain tandem repeats of the nucleotide sequence of the entire viral genome. Proc Natl Acad Sci U S A. 1978 Oct;75(10):4754–4758.

Thank you for bringing this omission to our attention. We have cited this manuscript, and refer to it explicitly:

LINE 172: "RNAs containing repetitive leader sequences were originally observed in MPyV by Acheson in 1978 [16] and subsequently described by others …"

Minor comment:

For the JC MT-like transcripts, are any of these predicted to contain transforming motifs of MuPyV MT?

We don't observe a MT-like transcript in JCPyV, but rather we observe it in BKPyV (see Figure 5B). The C-terminal region of the BK MT-like ORF is highly distinct from MPyV MT and has no detectable homology via comparison with BLASTP. Furthermore, the BK MT-like ORF does not have a predicted transmembrane domain.
